# Comment on: Toward an optimal contraception dosing strategy

**DOI:** 10.1371/journal.pcbi.1012398

**Published:** 2024-12-18

**Authors:** Giuseppe Benagiano, Andrea R. Genazzani, Giovanni Grandi, Sun-Wei Guo, Marwan Habiba

**Affiliations:** 1 Faculty of Medicine and Dentistry, ‘Sapienza’, University of Rome, Rome, Italy; 2 Geneva Foundation for Medical Education and Research, Geneva, Switzerland; 3 Division of Obstetrics and Gynecology, Department of Clinical and Experimental Medicine, The University of Pisa, Pisa, Italy; 4 Department of Medical and Surgical Sciences for Mother, Child and Adult, University of Modena and Reggio Emilia, Azienda Ospedaliero Universitaria Policlinico, Modena, Italy.aculty of Medicine, University of Modena, Modena, Italy; 5 Department of Biochemistry and Molecular Biology, Research Institute of Shanghai Obstetrics & Gynecology Hospital, Fudan University, Shanghai, China; 6 Department of Health Sciences, University of Leicester and University Hospitals of Leicester, Leicester, United Kingdom; University of Michigan, UNITED STATES OF AMERICA

## Abstract

The optimal contraception dosing strategy proposed by Gavina et al. recently is a commendable attempt to model a complex physiological process with potenial to apply to real-life data. However, there is a need to take into account the real challenges that arise when moving from the theory to its practical application, and it is important that lessons learnt from clinical studies be taken into consideration in any theoretical modelling. In this manuscript, we spell out these lessons and elaborate practical challenges facing their model.

We have read the report by Gavina et al [1] with great interest. In the article, the “*optimal control theory”* is applied to a modified menstrual cycle model with the aim of identifying the minimal daily dosages of the estrogen and progestin, individually or in combination, that are needed to block ovulation for contraceptive purposes. Indeed, although reproductive biologists are unlikely to be familiar with the mathematical model itself, they will probably welcome the objective of this work, and the application in complex biological models is commendable.

The starting point of this work has been developing a simplified menstrual cycle model which includes data centralization around the time of the LH surge at approximately mid-point of an idealized 28-day menstrual cycle. The model applied has been described by Harris in a 2002 thesis and further refined by Harris and Selgrade [1]. The model is then described as “*an autonomous nonlinear system of 13 delay differential equations* (DDEs) *merging the pituitary and ovarian components*”.

This is not the first effort in this emerging field, but the novelty of what proposed by Gavina et al [1] is the introduction of a modified mathematical model to identify the minimum total estrogen/progesterone dose, and timing of administration capable of inducing anovulation. Using this model, the authors raise the prospect of being able to optimize hormonal contraception by reducing the dose of steroids currently in use. The model posits that it is possible to predict the mean daily levels of LH and FSH, and of estradiol and progesterone throughout a physiological menstrual cycle and, as a consequence, to calculate the amount of reduction in these hormone levels in response to the administration of exogenous estrogen and/or progesterone.

When replicated in a biological system, their calculations indicate that it would be feasible to reduce–in a monotherapy application–the total estrogen dose by 92%, and by 43% that of progesterone. Their model also suggests that the most effective time for estrogen delivery for contraception is the mid follicular phase and that in a combined estrogen and progesterone regimen, the doses can be reduced ever further.

Gavina et al [1] propose that mathematical modelling “*may give clinicians insights into optimal formulations and schedule of therapy that can suppress ovulation*”.

It is a fact that, driven by the desire to reduce side effects, biologists and clinicians have endeavored to reduce the daily dose of the steroids administered. Specifically, over the last decades, reducing the estrogen daily dose has become a priority in view of its various side effects (e.g., [2]). This has been successfully achieved, since the daily dose of the estrogenic component (ethinyl-estradiol) has been reduced from an initial 75 to 50 μg [3] down to 20 μg [4].

In the case of the progestin component, given the variety of molecules utilized (e. g., [5]) the situation is more complex because, chemically, progestins used in contraception are derivatives of a variety of parent steroids (progesterone, 17α-hydroxy-progesterone, 19nor-progesterone, 19nor-testosterone, estrane, gonane, or spironolactone). Their progestogenic activity varies widely. Consequently, the daily dose required in a combined oral contraceptive (COC) varies from 75 μg [6] and 3 mg [7]---a full 40-fold difference. These synthetic steroids are capable of modulating progesterone receptors with a spectrum of activities. For this reason, besides the progesterone receptors, which mediate the action of these steroids, they bind with different affinities to receptors of other hormones, such as mineralocorticoid.

Given the situation, efforts to achieve a reduction in the daily dose of both components are laudable, while, at the same time, there is a need to take into account the real challenges that arise when moving from the theory to its practical application.

In this regard, it is important that lessons learnt from clinical studies be taken into consideration in any theoretical modelling. These include:

1. Menstrual cycles are highly variable both within and between individuals. The ideal 28-day menstrual cycle is rarely consistent in longitudinal follow-up studies of healthy women and the factors affecting inter- and intra-individual variability are poorly explained. It is unclear how this variation, even intra-individual, would impact the outcomes of mathematical modelling, raising the issue of how robust the model is in the face of such variations.
2. The variability in menstrual cycle affects the duration of both the follicular and the luteal phases. This renders detection of ovulation difficult, if not impossible.
3. Over an administration cycle, there is a wide day-to-day variation in the level of circulating steroids in healthy women. There is also a wide inter-individual variability.
4. There is a wide inter-individual variation in the serum level of administered steroids following oral intake. In addition, in the majority of cases serum levels peak within the first three hours following an oral dose. This complicates our understanding of the relation between serum levels and the desired clinical response (i. e., the inhibition of ovulation).
5. The contraceptive efficacy of steroidal contraception if not solely reliant on its ability to inhibit ovulation. There are recognized effects on the endometrium and on the cervical mucus which contribute to contraceptive efficacy of the steroids utilized today; these become especially useful in cases when ovulation is not suppressed.
6. Acceptability of a contraceptive method is dependent (amongst others) on good cycle control and the avoidance of breakthrough bleeding, both of which require adequate levels of steroids.

The proposed method has enormous potential in the identification of the “*minimal possible daily dose of both the estrogen and progestin components in a combined oral contraceptive capable to cause the inhibition of ovulation*” [1]. It is because of the importance of being able to apply this model to optimize hormonal contraception that we feel that clarification is necessary on several aspects of the proposed model.

Irrespective of what the model may indicate, decades of clinical experience have proved that COC need to be administered daily for a minimum of three weeks to obtain a reliable anti-fertility effect, and an acceptable cycle control [8]. In addition, other regimens have also been tested, including the continuous administration for 3 or 6 months [9].

A further point of caution should be raised when it comes to the proposed use of estrogen-only preparations. Progestin-only pills are effective because progestins alone are able to block ovulation, while maintaining an acceptable bleeding profile [10]. On the other hand, post-marketing surveillance of cyclical combined contraceptives that contained 21 days of estrogen and only 5 days of progestogens have shown a rise in the incidence of endometrial cancer and led to these being withdrawn after a relatively short period of use [11]. This is perhaps not surprising because inhibition of ovulation will leave the endometrium exposed to unopposed estrogen. The same problem was found when using estrogen-only post-menopausal replacement treatment [12].

Most importantly, while COC containing natural estrogens (i.e. micronized estradiol, estetrol) or an ester of estradiol (i.e., estradiol valerate) offer a valid alternative [13, 14], at present the great majority of products (around 95% of prescriptions) contain the synthetic estrogen ethinyl-estradiol (EE) [13]. In addition, the pharmacodynamic profile of the orally active EE differs from that of estradiol [15]. Similarly, in the case of the progestational component, literally dozens of *synthetic* molecules are utilized, each with its distinct spectrum of progestogenic as well as (anti) estrogenic and/or (anti) androgenic properties. Each of these compounds has its own pharmacodynamic profile that can also vary between individuals and is likely to exert significant changes in a woman’s physiology beyond the control of ovulation.

Given this enormous level of uncertainty and variability coupled with significant gaps in knowledge, it would appear that the concept of “personalizing” the administration of the two contraceptive steroids may be quite commendable, but, despite its appeal, seems rather premature.

On the other hand, currently available preparations offer a practical solution that has endured years of scrutiny by thousands of investigators and millions of users worldwide.

In clinical practice, what a COC must achieve is the inhibition of ovulation irrespective of the woman’s cycle length, by administering both components for a minimum of 21 days. This is necessary in order to maintain the “normal cycle length”, something considered important by a majority of users.

The present situation may be considered as “overdosing” compared to that envisaged mathematically, but it is a practical and proven way to proceed given the need to provide a standardized method accessible by millions of users.

It is for this reason that we consider it necessary to stimulate further refinement or perhaps an overhaul from the authors that draws on their unique expertise in mathematical modeling to find ways that apply to the “real life” situation.
